# Thermal History Dependent Al Distribution in Aluminum Substituted Strontium Hexaferrite

**DOI:** 10.3390/ma13040858

**Published:** 2020-02-13

**Authors:** Manuel Häßner, Denis A. Vinnik, Rainer Niewa

**Affiliations:** 1 University of Stuttgart, Pfaffenwaldring 55, 70569 Stuttgart, Germany; manuel.haessner@iac.uni-stuttgart.de; 2 South Ural State University, Lenin’s Prospect 76, Chelyabinsk 454080, Russia; denisvinnik@gmail.com

**Keywords:** cation distribution, aluminum substituted hexaferrite, magnetic materials

## Abstract

Single crystals of aluminum substituted strontium hexaferrite SrFe_12–*x*_Al*_x_*O_19_ were grown from sodium oxide based flux. The substitution level aimed for was *x* = 1.2. Annealing experiments performed on single crystals show that the Al distribution on the five iron sites of the hexaferrite structure depends on the annealing time at 900 °C. Single crystal X-ray diffractometry shows that annealing a crystal after the initial synthesis has an impact on the Al content on the octahedrally and tetrahedrally coordinated sites. Furthermore, it was found that heating in a corundum crucible increases the overall Al content. Magnetic measurements show that annealing in a platinum or corundum crucible decreases coercivity and remanence while the saturation magnetization is hardly influenced.

## 1. Introduction

The materials class of ferrites comprises a whole field of oxidic compounds with similar building motifs. They can all be described by stacking certain building blocks, called S, R and T blocks [1]. Although a wide variety of ferrites is known, the best-known ones are certainly the spinel (also called S-type) [2,3] and the barium hexaferrite BaFe_12_O_19_ (called M-type, BaM) [4]. The M-type ferrite shines with superior magnetic properties, relatively high Curie temperatures and good chemical stability. These properties grant it several applications in the industry such as in storing components, part of inductors and in microwave devices [5,6] and make the M-type a prominent topic in functional materials research. Several synthesis methods have been described like the classic solid-state synthesis, crystal growth in flux, sol-gel method and hydrothermal approaches to receive the product in bulk, as thin films or even as nanoparticles [7,8,9,10,11,12,13]. Many researchers focus on substituting iron, barium or both and the resulting influence on the structure and properties. Substitutions with various isovalent cations have been reported, like strontium(II) and lead(II) for barium(II) [14,15] as well as aluminum(III) and chromium(III) for iron(III) [16,17]. Furthermore, aliovalent substitutions are possible, such as lanthanum(III) [18] on the alkaline earth metal site and copper(II), titanium(IV) or tungsten(VI) for the transition metal [19,20,21]. The latter kind of substitution can be accomplished in higher degree by co-substitution with ions carrying higher and lower charges than the substituted element. In the case of manganese substitution, three different oxidation states Mn(II), Mn(III) and Mn(IV) were detected to coexist via X-ray absorption spectroscopy [22].

We have studied Al(III) substituted strontium hexaferrite, with a special focus on the cation distribution on the mixed sites and resulting magnetic properties. Introducing Al(III) to the hexaferrite system is reported to increase the coercivity, which is desirable for many applications. Saturation magnetization and remanence decrease concurrently, as aluminum(III) has no unpaired spins as opposed to iron(III) [23]. Using the knowledge accumulated by investigating hexaferrites with different substituents and degrees of substitution, the magnetic properties of the material can be fine-tuned to fit its desired use best.

## 2. Materials and Methods 

All used chemicals were obtained from the Ural Plant of Chemicals, Russia, with a purity of 99.9%. Aluminum substituted M-type ferrite SrFe_12−*x*_Al*_x_*O_19_ (SrM) was prepared from strontium carbonate (SrCO_3_), iron(III) oxide (γ-Fe_2_O_3_) and aluminum oxide (γ-Al_2_O_3_) with the molar ratio 1:5.4:0.6. The initial mixture contained 26.3 mol-% of sodium carbonate (Na_2_CO_3_) as flux to enhance crystal growth during the procedure, as Gambino and Leonhard suggested in their work [24]. The powder mixture was homogenized using an agate mortar and then filled into a platinum crucible. The crucible was heated in a resistance furnace, which was equipped with a thermocouple PR-30/6 as well as a precision thermostat RIF-101. For further homogenization in the flux, the mixture was held at 1260 °C for 3 h and then cooled to 900 °C with a cooling rate of 4.5 K/h. Afterwards, the system was allowed to cool to room temperature naturally.

The synthesis yielded a crystalline product with crystal sizes up to several millimeters. Selected crystals were annealed to examine any cation distribution changes in the structure. A larger crystal was selected and divided in three pieces. One was put in a platinum crucible and one in a corundum crucible and heated to 900 °C. The temperature was held for 20 h followed by a slow cooling with a rate of 15 K/h. Various fragments of these crystals were analyzed via single crystal X-ray diffraction (scXRD) and wavelength dispersive X-ray spectroscopy (WDX).

Powder X-ray diffraction was carried out using a STADI-P diffractometer from STOE&Cie equipped with a Mythen-1K detector and Mo *K_α_* radiation (*λ*_Mo_ = 71.073 pm). ScXRD was performed on smaller fragments with a four-circle diffractometer *κ*-CCD by Bruker-Nonius with monochromatic Mo *K*_α_ radiation.

The composition and homogeneity of several crystals were further investigated via scanning electron microscopy (SEM) and WDX using a Cameca SX 100. The samples were coated with a thin carbon layer to avoid accumulation of charge. Used standards for the quantification were celestine (SrSO_4_) for strontium, hematite (Fe_2_O_3_) for iron and corundum (Al_2_O_3_) for aluminum.

Magnetic properties were measured using a MPMS3 SQUID magnetometer from Quantum Design, allowing hysteresis measurements with a magnetic field strength range of −70 kOe ≤ *H* ≤ 70 kOe. The determined properties were coercivity, remanence and saturation magnetization. The powdered sample or selected crystals were either put in plastic capsules and immobilized using cotton wool or fixed on a rotatable sample holder with a special glue. 

## 3. Results and Discussion

### 3.1. Structure and Composition

**Figure 1:**
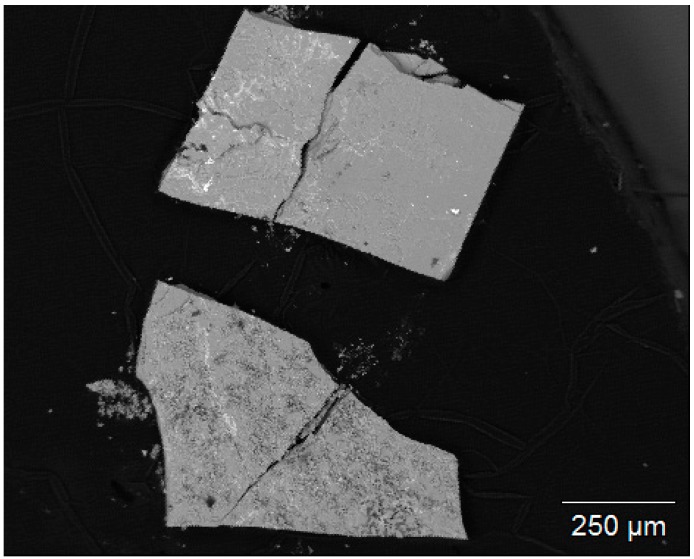
Backscattered electron scanning electron microscope (SEM) picture of two exemplary crystals, which were investigated via wavelength dispersive X-ray spectroscopy (WDX).

**Figure 2:**
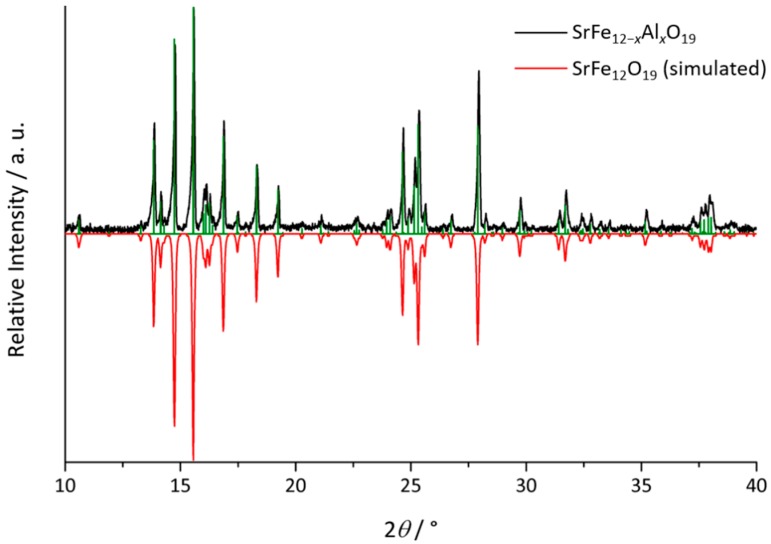
Powder diffraction pattern of SrFe_12−*x*_Al*_x_*O_19_ (black, up) with marked position of reflexes (green) and simulated pattern of unsubstituted SrFe_12_O_19_ (red, down [14]) measured using Mo-*K*_α_ radiation.

Figure 1 shows a backscattered electron SEM picture of two exemplary crystals of up to about 800 µm length. The powder diffraction pattern of the product (black, up), and a simulated diffractogram of unsubstituted strontium hexaferrite SrFe_12_O_19_ (SrM, red, down [14]) as references, are shown in Figure 2. A comparison of both patterns proves that the described synthesis yields single phase material. It can also be seen that minor Al substitution hardly affects reflex positions and intensities compared to unsubstituted SrM.

Both the powder pattern and the single crystal data refinement show that an aluminum containing product, like unsubstituted SrM, crystallizes in the magnetoplumbite structure [4,14], space group *P*6_3_/*mmc* with *a* ≈ 587 pm and *c* ≈ 2300 pm. The exact lengths of the unit cell axes vary depending on Al content and annealing time. Crystallographic data, refinement parameters and measurement conditions for three investigated single crystals with different thermal history are summarized in Table 1. Crystal 1 was investigated directly after the synthesis. Crystals 2 and 3 were annealed for 20 h at 900 °C in platinum or corundum crucibles, respectively.

**Figure 3:**
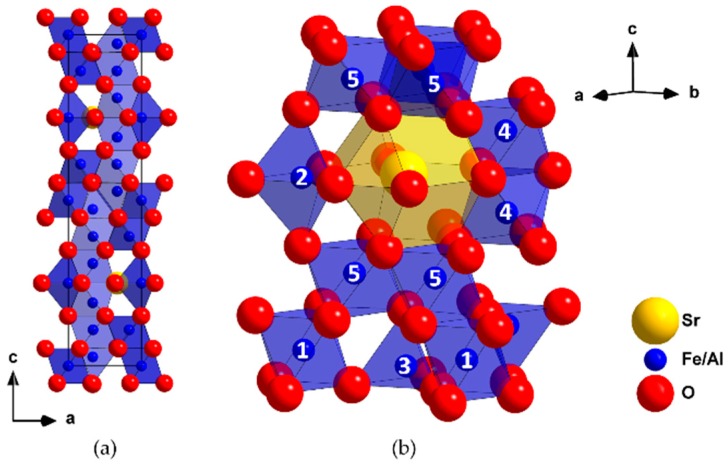
(**a**) Unit cell of the product with magnetoplumbite structure, viewed along [010] with polyhedra around *M*; (**b**) A section of the hexagonal structure where iron sites are labelled with numbers 1 (*M*(1), octahedrally coordinated), 2 (*M*(2), trigonal bipyramidally coordinated), 3 (*M*(3), tetrahedrally coordinated), 4 and 5 (*M*(4), *M*(5), octahedrally coordinated) and are mixed occupied by both iron and aluminum in different ratios.

The crystal structure of the magnetoplumbite SrFe_12_O_19_ is built from close packed oxygen or oxygen-strontium layers which are stacked in both cubic (S-block) and hexagonal sequence (R-block) and can be described as [BAB’ABCAC’AC]*_n_*. One fourth of oxygen anions are replaced by strontium cations in B’ and C.’ Iron occupies five crystallographically unique sites in the lattice, with three of them being octahedrally, and the remaining two being tetrahedrally and trigonal-bipyramidally coordinated, respectively [4,14]. The extended unit cell of the magnetoplumbite structure is depicted in Figure 3 (left), as well as a section of the structure with labelled mixed sites and coordination polyhedra around the cations, where *M* = Fe, Al (right). 

The literature states different favored cation sites for Al in the magnetoplumbite structure. Both Albanese and Choi et al. investigated BaM via Mößbauer spectroscopy in 1995 and 2004 respectively. Examining the quadrupole splitting of Al substituted barium hexaferrite they found that Al preferably occupies *M*(5) as well as *M*(1) and *M*(3) to a smaller degree [25,26]. In an earlier work, Albanese et al. described that occupation of *M*(2) by Al in SrM is even less likely [27]. Contrary to this, Awawdeh et al. stated in 2014 that *M*(4) is preferred, using the same method [28]. Vinnik et al. investigated Al substituted BaM using scXRD. According to the refinement, Al prefers *M*(1) and then *M*(5) with smaller amounts on *M*(2) and *M*(4). The tetrahedrally coordinated *M*(3) is not occupied at small Al contents around *x* = 1 [16]. In our scXRD refinements on crystals obtained after the initial synthesis, we could confirm the general results of Vinnik. *M*(3) has little to no Al but we found that the majority of Al is located on *M*(5). To investigate these reported differences in the occupation, annealing experiments of single crystals were performed. During the investigation via WDX and refinement of scXRD data, it became evident that the Al distribution on the trivalent metal cation sites and the homogeneity of the received crystals are dependent on the synthesis parameters like annealing time. The asymmetric unit for every unique ion site in the structure as well as the distribution of Al on the iron sites and the isotropic displacement factors *U*_iso_ for a crystal obtained in the initial synthesis are given in Table 2. The same is shown in Table 3 for a crystal annealed in a Pt crucible and in Table 4, for a crystal annealed in a corundum crucible.

After annealing in a platinum or a corundum crucible, redistribution was indeed observed in occupation of the mixed sites. As can be taken from Table 2, Table 3 and Table 4, the coordinates of the ions did not change significantly and the *U*_iso_ are similar before and after the annealing experiments. The aluminum distribution however did change. Upon annealing, its content is doubled on the octahedrally coordinated site *M*(1) whereas it is significantly reduced on *M*(3), while the refined substitution levels are similar. The contents on *M*(2) and *M*(4) in the R-block are affected to only a small degree. Noteworthy, upon annealing, the unit cell parameters shrinks by about 0.2% leading to a density increase of 0.5% due to atomic redistribution.

According to the refinement, annealing in a corundum crucible slightly increases the Al content in the structure. WDX experiments yielded additional information about the homogeneity and Al content of the material. Crystals with edge lengths up to 1 mm, taken from the initial synthesis, after annealing in a platinum crucible or in a corundum crucible, were examined at six to ten locations on the crystal surface. The average Al contents in at.-% for the three samples are shown in Table 5. The errors represent the highest discrepancy of a single value to the average. The high errors of the former two samples indicate that, although the Al distribution on the different metal ion sites changes, annealing alone will not suffice to homogenize the material. However, it is possible that additional Al ions can diffuse into the hexaferrite when heated while in contact with the corundum crucible and a homogeneous distribution can be achieved in this way. This can be derived from the lower error of the average content after annealing in the corundum crucible. It can also be seen that the relative content of Al increases in comparison to that obtained directly after the initial synthesis and annealing, which both took place in a platinum crucible. This strengthens the presumption that Al diffuses into the crystal from the crucible material. This is very probable as corundum and hematite crystallize isotypic [29].

### 3.2. Magnetism

**Figure 4:**
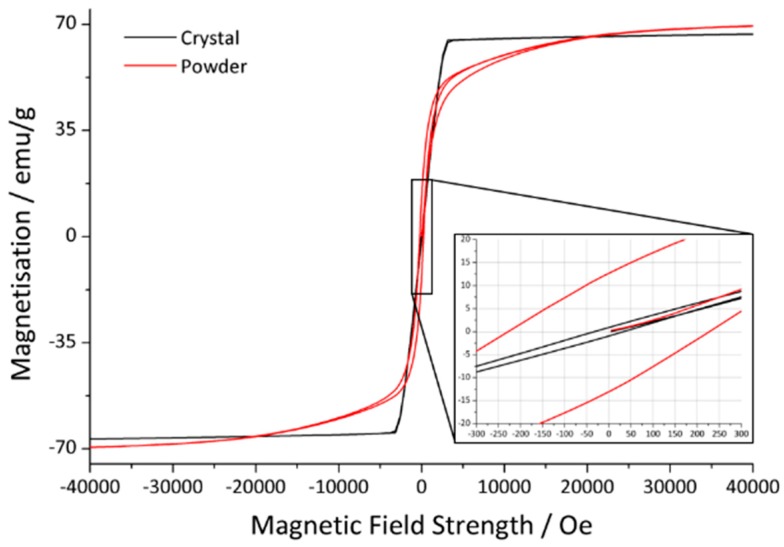
Hysteresis curve of a crystal (black) and a powder sample (red). The zoomed in part emphasizes the coercivity and the remanence.

**Figure 5:**
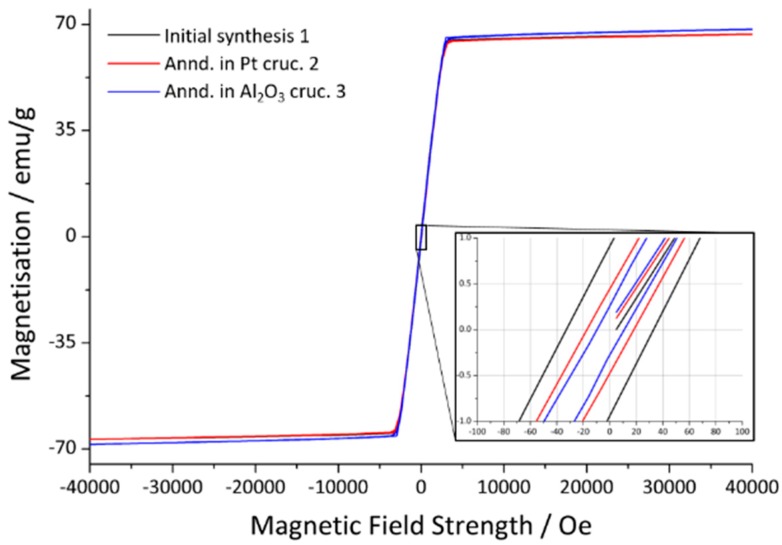
Hysteresis curves of a crystal sample from the initial synthesis (black), after annealing in a platinum crucible (red) and corundum crucible (blue) with the magnetic field applied parallel to the *c* axis. The area around the origin is magnified to show the coercivity and remanence.

Magnetic measurements were performed on both powdered samples and larger crystals. The magnetic field was applied along the *c* axis for the latter. In Figure 4 and Figure 5, the magnetic moment is plotted against the magnetic field strength to investigate the magnetic hysteresis. The former figure shows a curve for a powder (red) and a crystal (black). It can be seen that the hysteresis of the crystal is much steeper than the hysteresis of the powder, which is desired in a hard magnetic material but widens considerably in a powdered sample.

The magnetic hysteresis loops for three crystals after different degrees of annealing, as described in Section 2, are shown in Figure 5. The magnetic data derived from the curves, the saturation magnetization *M*_s_, remanence *M*_r_ and coercivity *H*_c_, are summarized in Table 6. It is obvious that the hysteresis shape and *M*_s_ do not vary much, whereas *M*_r_ and *H*_c_ decrease significantly after annealing.

According to the literature, both the saturation magnetization and remanence decrease significantly and the coercivity increases with higher substitution rates [26,28,30,31]. El-Sayed et al. report a drop of *M*_s_ from 61.2 emu/g to 49.7 emu/g, *M*_r_ from 31.5 emu/g to 25.8 emu/g and an increase in *H*_c_ from 1.66 kOe to 1.77 kOe [30]. However, those values highly depend on the synthesis temperature and particle size [30,31]. The relatively low values presented here are to be expected as plates in millimeter scale were investigated. The critical domain size is exceeded, which results in lower overall values [31]. A drop of *M*_r_ was indeed observed after annealing concomitant to a decreasing *H*_C_ (see Table 6).

## 4. Conclusions

Single crystals of aluminum substituted strontium hexaferrite, SrFe_12–*x*_Al*_x_*O_19_, up to several millimeters in length, were grown from a sodium oxide based flux in a platinum crucible. ScXRD measurements show that the aluminum distribution over the five available sites depends on the duration of the annealing. The WDX measurements show that previously inhomogeneous Al substituted hexaferrites can be homogenized by annealing in a corundum crucible, while slightly increasing the degree of substitution. These results offer a direct explanation for various contradicting reports on metal ion distributions in hexaferrites. The shape of the magnetic hysteresis loop and the *M*_s_ value are hardly affected by the annealing but *M*_r_ and *H*_c_ decrease when annealing after the initial synthesis. The powder sample shows a broader hysteresis curve.

## Figures and Tables

**Table 1:** Comparison of crystallographic data, refinement parameters and measurement conditions for crystals treated under different conditions.

Crystal	1	2	3
Refined composition	SrFe_11.2(1)_Al_0.8(1)_O_19_	SrFe_11.2(2)_Al_0.8(2)_O_19_	SrFe_11.0(1)_Al_1.0(1)_O_19_
Crystal system	Hexagonal		
Space group type	*P*6_3_/*mmc* (No. 194)		
*a*/pm	588.37(1)	587.39(1)	587.41(1)
*c*/pm	2304.34(7)	2300.42(6)	2300.62(6)
*Z*	2	2	2
Density *ρ* (calculated)/ g/cm³	4.966	4.991	4.990
Volume *V*/10^8^·pm³	6.9084(3)	6.8737(2)	6.8748(2)
Diffractometer	*κ*-CCD (Bruker-Nonius)		
Wavelength	Mo-*K*_α_, *λ* = 71.073 pm		
Index ranges *hkl*	*h* = ±8; *k* = ±9; *l* = ±36	–8 ≤ *h* ≤ 7; k = ±9; –21 ≤ *l* ≤ 36	*h* = ±9; *k* = ±9; –34 ≤ *l* ≤ 36
Measurement range *θ*_max_/°	69.88	69.87	69.87
*F*(000)	978.0	978.0	978.0
*µ*(Mo K*_α_*)/mm^–1^	15.18	15.26	15.25
Reflections collected/independent	15718/645	14639/645	16598/645
*R*_int_/*R_σ_*	0.0445/0.0151	0.0620/0.0193	0.0733/0.0225
*R*_1_/*R*_1_ with *F*_0_ ≥ 4*σ*(*F*_0_)	0.0297/0.0245	0.0371/0.0310	0.0443/0.0324
*wR*_2_/*GooF*	0.0576/1.090	0.0785/1.155	0.0872/1.125
Largest e^−^ difference peak/hole	0.89/–0.82	0.85/–1.49	1.27/–1.53

**Table 2:** Wyckoff sites, fractional coordinates in the asymmetric unit, occupational parameters and isotropic displacement factors for every unique ion site of the pristine crystal 1 (SrFe_11.2(1)_Al_0.8(1)_O_19_) with *M* = Fe, Al (composition from single crystal X-ray diffraction (scXRD) refinement).

Atom	Site	*x*/*a*	*y*/*b*	*z*/*c*	Occupation	*U*_iso_/pm^2^
Sr	2*d*	2/3	1/3	1/4	1	0.0144(2)
*M*(1)	2*a*	0	0	0	88/12(1)	0.0070(3)
*M*(2)	2*b*	0	0	1/4	92/8(2)	0.0138(3)
*M*(3)	4*f*	1/3	2/3	0.02729(3)	96/4(1)	0.0065(2)
*M*(4)	4*f*	1/3	2/3	0.19075(3)	93/7(1)	0.0070(2)
*M*(5)	12*k*	0.33750(8)	*x*/2	0.10914(2)	93/6(1)	0.0071(2)
O(1)	4*e*	0	0	0.1511(2)	1	0.0087(6)
O(2)	4*f*	2/3	1/3	0.0553(2)	1	0.0099(6)
O(3)	6*h*	0.1816(3)	2*x*	1/4	1	0.0119(6)
O(4)	12*k*	0.15616	2*x*	0.05231(8)	1	0.0085(4)
O(5)	12*k*	0.546(2)	$\bar{x}$	0.15080(8)	1	0.0097(4)

**Table 3:** Wyckoff sites, fractional coordinates in the asymmetric unit, occupational parameters and isotropic displacement factors for every unique ion site of crystal 2 after annealing in a platinum crucible (SrFe_11.2(2)_Al_0.8(2)_O_19_) with *M* = Fe, Al (composition from scXRD refinement).

Atom	Site	*x*/*a*	*y*/*b*	*z*/*c*	Occupation	*U*_iso_/pm^2^
Sr	2*d*	2/3	1/3	1/4	1	0.0152(3)
*M*(1)	2*a*	0	0	0	78/22(2)	0.0070(4)
*M*(2)	2*b*	0	0	1/4	93/7(2)	0.0151(4)
Fe(3)	4*f*	1/3	2/3	0.02729(3)	1	0.0071(3)
*M*(4)	4*f*	1/3	2/3	0.19056(4)	95/5(2)	0.0076(3)
*M*(5)	12*k*	0.33725(9)	*x*/2	0.10901(2)	94/6(1)	0.0077(2)
O(1)	4*e*	0	0	0.1507(2)	1	0.0082(7)
O(2)	4*f*	2/3	1/3	0.0558(2)	1	0.0092(8)
O(3)	6*h*	0.1816(4)	2*x*	1/4	1	0.0128(7)
O(4)	12*k*	0.1558(3)	2*x*	0.0521(1)	1	0.0084(5)
O(5)	12*k*	0.5043(3)	$\bar{x}$	0.1502(1)	1	0.0091(4)

**Table 4:** Wyckoff sites, fractional coordinates in the asymmetric unit, occupational parameters and isotropic displacement factors for every unique ion site of crystal 3 after annealing in an aluminum oxide crucible (SrFe_11.0(1)_Al_1.0(1)_O_19_) with *M* = Fe, Al (composition from scXRD refinement).

Atom	Site	*x*/*a*	*y*/*b*	*z*/*c*	Occupation	*U*_iso_/pm^2^
Sr	2*d*	2/3	1/3	1/4	1	0.0167(3)
*M*(1)	2*a*	0	0	0	76/24(2)	0.0077(4)
*M*(2)	2*b*	0	0	1/4	92/8(2)	0.0159(5)
*M*(3)	4*f*	1/3	2/3	0.02731(4)	98/2(1)	0.0079(3)
*M*(4)	4*f*	1/3	2/3	0.19058(4)	94/6(1)	0.0083(3)
*M*(5)	12*k*	0.33715(9)	*x*/2	0.10902(2)	92/8(1)	0.0083(2)
O(1)	4*e*	0	0	0.1508(2)	1	0.0095(8)
O(2)	4*f*	2/3	1/3	0.0555(2)	1	0.0097(9)
O(3)	6*h*	0.1817(4)	2*x*	1/4	1	0.0129(8)
O(4)	12*k*	0.1553(3)	2*x*	0.0521(2)	1	0.0099(5)
O(5)	12*k*	0.5040(3)	$\bar{x}$	0.1502(2)	1	0.0105(5)

**Table 5:** Average aluminum contents of samples from the initial synthesis and annealed samples in at.-%. Shown errors represent the highest discrepancy between a single value and the average.

Sample	Average Aluminum Content/at.-%
Crystal 1 from initial synthesis	1.7 ± 0.2
Crystal 2 from annealing in platinum crucible	2.0 ± 0.5
Crystal 3 from annealing in corundum crucible	2.15 ± 0.08

**Table 6:** Saturation magnetization *M*_s_, remanence *M*_r_ and coercivity *H*_c_ of four investigated samples.

Sample	*M*_s_/emu/g	*M*_r_/emu/g	*H*_c_/Oe
Powder	71.1	12.8	226.1
Crystal 1 from initial synthesis	67.8	0.9	33.0
Crystal 2 from annealing in platinum crucible	68.1	0.4	18.1
Crystal 3 from annealing in corundum crucible	69.9	0.3	10.9
